# Supplementary material for: Transcriptome Studies Reveal the N6-Methyladenosine Differences in Testis of Yaks at Juvenile and Sexual Maturity Stages
Source: Animals (Basel). 2023 Sep 5;13(18):2815. doi: 10.3390/ani13182815 (PMC10525320; doi:10.3390/ani13182815)
Supplement: Supplementary file 1 [file animals-13-02815-s001.zip › File S1.docx]

**Transcriptome studies reveal the regulation of m^6^A in testis development and spermatogenesis in yak**

**Shaoke Guo^1,2^, Jie Pei^1,2^, Xingdong Wang^1,2^, Mengli Cao^1,2^, Lin Xiong^1,2^, Yandong Kang^1,2^, Ziqiang Ding^1,2^, Yongfu La^1,2^, Min Chu^1,2^, Pengjia Bao^1,2^, Xian Guo^1,2*^**

^1^ Key Laboratory of Yak Breeding Engineering of Gansu Province, Lanzhou Institute of Husbandry and Pharmaceutical Sciences, Chinese Academy of Agricultural Sciences, Lanzhou, 730050, China.

^2^ Key Laboratory of Animal Genetics and Breeding on Tibetan Plateau, Ministry of Agriculture and Rural Affairs, Lanzhou, 730050, China.

gsk1125@163.com (S.G.); peijie@caas.cn (J.P.); wxd17339929758@163.com (X.W.); caomengliaaa@163.com (M.C.); xionglin@caas.cn (L.X.); kangyandong0901@163.com (Y.K.); dingziqiang1997@163.com (Z.D.); layongfu@caas.cn (Y.L.); chumin@caas.cn (M.C.); baopengjia@caas.cn (P.B.)

*Correspondence: guoxian@caas.cn; Tel.: +86-18993037854

**Materials and Methods**

**RNA extraction and cDNA synthesis**

The tissue preserved in liquid nitrogen was taken out and quickly ground into powder in a heat-sterilized mortar with liquid nitrogen. An appropriate amount of ground tissue was placed in a sterilized Eppendorf tube, and the total RNA of the tissue was extracted using TRIzol lysis reagent. NanoDrop ND-1000 (Thermo Fisher Scientific, CA, USA) was used to control the quantity and purity of RNA. Concentration >50 ng/μL, RIN >7.0, OD_260_/_280_ >1.8, and total RNA >50 μg met the requirements of downstream experiments. RNA was reverse transcribed according to the PrimeScript RT Reagent with the gDNA Eraser Perfect Real Time Kit (TaKaRa, Beijing, China) to obtain cDNA, which was frozen at −80°C for subsequent experiments.

**Quantitative detection of m^6^A levels and methylation-related enzymes**

According to the EpiQuik™ m^6^A RNA methylation quantitative detection kit (colorimetric) (Epigentek, NY, USA), the methylation level of m^6^A RNA in total RNA of testicular tissue was detected according to the steps specified by the supplier. METTL3, METTL14, WTAP, FTO, ALKBH5, YTHDF1-3, YTHDC1-2, RBM15, and VIRMA were selected as m^6^A-related enzymes. qRT-PCR was performed using SYBR Green Master Mix (Yeasen, Shanghai, China) and the LightCycler system (CFX96TM, Singapore). The primers of the target gene were synthesized using the NCBI website. The reaction volume was 20 μL, including 10 μL 2 × Precision PLUS Master Mix, 1 μL (25 ng) diluted cDNA, 1 μL (300 nmol each) gene-specific forward and reverse primers, and 7 μL RNase-free ddH_2_O. PCR conditions were as follows: 95°C for 3 min, 95°C for 10 s, 55°C for 30 s, 39 cycles. Data were expressed as mean ± standard error (n = 3). The glyceraldehyde-3-phosphate dehydrogenase gene was used as an internal reference gene, and the 2^−ΔΔCT^ calculation method was used to standardize mRNA expression. All statistical analyses were performed using SAS 9.4 statistical software, and *P* < 0.05 was considered statistically significant.

**Library construction and sequencing**

The qualified RNA samples were constructed into a cDNA library, and oligo-dT magnetic beads were used to specifically capture mRNA with polyadenylate by two rounds of purification. A magnesium ion interruption kit (NEBNext Magnesium RNA Fragmentation Module; New England Biolabs, MA, USA) was used for fragmentation of captured mRNA at 86°C for 7 min. The fragmented mRNA was pre-mixed with the immunomagnetic beads Dynabeads Antibody Coupling Kit (Thermo Fisher Scientific) and m^6^A Antibody (Synaptic Systems, Germany) in immunoprecipitation (IP) buffer (50 mM Tris-HCl, 750 mM NaCl, and 0.5% Igepal CA-630) before conducting IP. The IP product was passed through reverse transcriptase (Invitrogen SuperScript™ II Reverse Transcriptase, CA, USA). Then *E. coli* DNA polymerase I was combined with RNase H (New England Biolabs) and mixed with dUTP solution (Thermo Fisher Scientific) for two-strand synthesis and RNA degradation. AMPure XP beads were used to select the fragment size, and a 300 ± 50 bp library was obtained. Finally, two-terminal sequencing was performed on the Illumina NovaSeq™ 6000 platform (Illumina Inc., San Diego, CA, USA) with sequencing mode PE150.

**Bioinformatics analysis**

Trimmomatic [1] software was used to trim the raw reads of the generated FASTQ format sequence, filtering out low-quality and N bases or low-quality reads, and obtaining high-quality clean reads. A total of 250,000 pairs of reads were randomly selected from the clean reads after quality control, and compared with the nucleotide library using the BLASTN software. SortMeRNA [2] software was used to remove ribosomal RNA. The clean reads after ribosomal RNA removal were used for subsequent genome alignment analysis. The default parameters of HISAT2 [3] software were used to compare clean reads to the yak reference genome (LU_Bosgru_v3.0), and only aligned reads were retained for subsequent analysis. To verify the efficiency of the MeRIP-seq experiment, Guitar [4] R package and deepTools [5] software were used for quality inspection of genome comparison result data. MeTDiff [6] software was used to perform peak detection with input samples as control. The detected peak was annotated with ChIPseeker [7] software. MEME [8] and DREME [9] software was used to detect motifs in peak sequences. Simultaneously, Tomtom software was used to compare the obtained motif sequence with the known motif database, and the known motif was used in annotation differential analysis to identify RNA methylation in the experimental–control group study. The differential peak was detected using MeTDiff software, and the detected differential peak was annotated using ChIPseeker. For the peak and differential peak detected previously, GO (http://geneontology.org/) and KEGG (http://www.genome.jp/kegg/) enrichment analyses were conducted by the hypergeometric distribution test to determine the biological function or pathway that the peak gene mainly affects.

**Reference**

1. Bolger, A. M.; Lohse, M.; Usadel, B., Trimmomatic: a flexible trimmer for Illumina sequence data. *Bioinformatics (Oxford, England)* **2014,** 30, (15), 2114-20.

2. Kopylova, E.; Noé, L.; Touzet, H., SortMeRNA: fast and accurate filtering of ribosomal RNAs in metatranscriptomic data. *Bioinformatics (Oxford, England)* **2012,** 28, (24), 3211-7.

3. Kim, D.; Langmead, B.; Salzberg, S. L., HISAT: a fast spliced aligner with low memory requirements. *Nature methods* **2015,** 12, (4), 357-60.

4. Cui, X.; Wei, Z.; Zhang, L.; Liu, H.; Sun, L.; Zhang, S. W.; Huang, Y.; Meng, J., Guitar: An R/Bioconductor Package for Gene Annotation Guided Transcriptomic Analysis of RNA-Related Genomic Features. *BioMed research international* **2016,** 2016, 8367534.

5. Ramírez, F.; Dündar, F.; Diehl, S.; Grüning, B. A.; Manke, T., deepTools: a flexible platform for exploring deep-sequencing data. *Nucleic acids research* **2014,** 42, (Web Server issue), W187-91.

6. Cui, X.; Zhang, L.; Meng, J.; Rao, M. K.; Chen, Y.; Huang, Y., MeTDiff: A Novel Differential RNA Methylation Analysis for MeRIP-Seq Data. *IEEE/ACM transactions on computational biology and bioinformatics* **2018,** 15, (2), 526-534.

7. Yu, G.; Wang, L. G.; He, Q. Y., ChIPseeker: an R/Bioconductor package for ChIP peak annotation, comparison and visualization. *Bioinformatics (Oxford, England)* **2015,** 31, (14), 2382-3.

8. Bailey, T. L.; Boden, M.; Buske, F. A.; Frith, M.; Grant, C. E.; Clementi, L.; Ren, J.; Li, W. W.; Noble, W. S., MEME SUITE: tools for motif discovery and searching. *Nucleic acids research* **2009,** 37, (Web Server issue), W202-8.

9. Schulz, M. H.; Devanny, W. E.; Gitter, A.; Zhong, S.; Ernst, J.; Bar-Joseph, Z., DREM 2.0: Improved reconstruction of dynamic regulatory networks from time-series expression data. *BMC systems biology* **2012,** 6, 104.
